# Patterns of invasive recurrence among patients originally treated for ductal carcinoma in situ by breast-conserving surgery versus mastectomy

**DOI:** 10.1007/s10549-021-06129-3

**Published:** 2021-03-06

**Authors:** Kate R. Pawloski, Audree B. Tadros, Varadan Sevilimedu, Ashley Newman, Lori Gentile, Emily C. Zabor, Monica Morrow, Kimberly J. Van Zee, Laurie J. Kirstein

**Affiliations:** 1 Breast Service, Department of Surgery, Memorial Sloan Kettering Cancer Center, New York, NY USA; 2 Biostatistics Service, Department of Epidemiology and Biostatistics, Memorial Sloan Kettering Cancer Center, New York, NY USA; 3 Breast Service, Department of Surgery, Memorial Sloan Kettering Cancer Center, 480 Red Hill Road, Middletown, NJ 07748 USA

**Keywords:** Ductal carcinoma in situ, Breast-conservation surgery, Mastectomy, Invasive recurrence, Breast cancer

## Abstract

**Purpose:**

Local recurrence after treatment of ductal carcinoma in situ (DCIS) with breast-conserving surgery (BCS) is more common than after mastectomy, but it is unclear if patterns of invasive recurrence vary by initial surgical therapy. Among patients with invasive recurrence after treatment for DCIS, we compared patterns of first recurrence between those originally treated with BCS vs. mastectomy.

**Methods:**

From 2000 to 2016, women with an invasive recurrence occurring ≥ 6 months after initial treatment for DCIS were retrospectively identified. Clinicopathologic features and adjuvant treatment of the initial DCIS, as well as characteristics of first invasive recurrences, were compared between patients who had undergone BCS vs. mastectomy.

**Results:**

452 patients with an invasive recurrence after surgery for DCIS were identified: 367 patients (81%) had initially undergone BCS and 85 patients (19%) mastectomy. Patients originally treated with mastectomy were younger and were more likely to have had high grade, necrosis, and multifocal or multicentric DCIS (*p* < 0.001) compared with the BCS group. A higher proportion of invasive recurrences were local after BCS (93%; 343/367), whereas 88% (75/85) of recurrences after mastectomy were regional or distant (*p* < 0.001). The median time to first invasive recurrence was not different between surgical groups (BCS: 6.4 years vs. mastectomy: 5.5 years; *p* = 0.12).

**Conclusions:**

Among women who experienced a first invasive recurrence after treatment for DCIS, those who had originally undergone mastectomy more commonly presented with advanced disease compared to those treated with BCS, likely related to the absence of the breast and the higher risk profile of their initial DCIS.

**Supplementary Information:**

The online version of this article (10.1007/s10549-021-06129-3) contains supplementary material, which is available to authorized users.

## Introduction

Local recurrence after breast-conserving surgery (BCS) for ductal carcinoma in situ (DCIS) is more common than after mastectomy (13–25% vs. 3% after 10 years); however, 10-year breast cancer-specific mortality is low regardless of surgical therapy (BCS: 1.9–2.0% vs. mastectomy: 1.3%) [1]. Of all local recurrences following BCS, approximately half are DCIS and half are invasive, whereas most recurrences after mastectomy are invasive [1, 2]. We sought to compare women with invasive recurrence after treatment for DCIS, to assess patterns of first recurrence and characteristics of initial DCIS among patients originally treated with BCS vs. mastectomy. We did not examine all patients with DCIS, only those with an invasive recurrence (local, regional or distant), therefore recurrence and mortality rates were not assessed.

## Methods

Following Institutional Review Board approval, we retrospectively identified patients treated for an invasive recurrence from 2000 to 2016 at our institution following treatment for DCIS. All invasive recurrences were treated at our institution; however, patients were included regardless of the site of treatment of the initial DCIS.

Clinicopathologic and treatment factors pertaining to the initial DCIS, and details of invasive recurrences were recorded. DCIS grade was ascertained from original pathology reports and was classified as high, intermediate, or low. Multifocality and multicentricity were determined from pathology and radiology reports; multifocality was defined as DCIS in multiple areas of the same quadrant of the breast and multicentricity was defined as disease in separate quadrants. Margins were categorized as negative, close (≤ 2 mm) or positive (tumor on ink). Patients who underwent sentinel lymph node biopsy (SLNB) or axillary lymph node dissection (ALND) were categorized as having undergone axillary surgery.

Invasive recurrence was defined as having occurred ≥ 6 months after initial treatment for DCIS. Recurrence subtype was categorized by hormone receptor (HR) and HER2 status (HR+/HER2−, HR+/HER2+, HR−/HER2+, HR−/HER2−) as assessed by immunohistochemistry and fluorescence in situ hybridization. Histology of the invasive recurrence was categorized as ductal, lobular, or other, which included mixed ductal/lobular or mammary carcinoma. Recurrences were classified as local (in-breast following initial BCS or ipsilateral chest wall following initial mastectomy), regional (ipsilateral axillary or supraclavicular lymph nodes), or distant. Although no staging guidelines exist for invasive recurrence after DCIS, for ease of comparison, clinical and TNM staging of the recurrences were classified using the 7th Edition of the American Joint Committee on Cancer guidelines [3].

Continuous variables were summarized by median and range, and between-group differences were assessed using the Wilcoxon rank-sum test. Categorical variables were summarized by frequency and percentage and were compared between groups using Chi-square or Fisher’s exact test. All patients in this series experienced an invasive recurrence, obviating the need to adjust for loss of follow-up, a priori, in a time-to-event analysis. Between-group differences in crude time to invasive recurrence were assessed using an accelerated failure time model [4] to adjust for initial DCIS characteristics and treatment, including age at surgery, type of initial surgery, multifocality/multicentricity, grade, presence of necrosis, histology, margin status, receipt of endocrine therapy or radiation. Kaplan–Meier methods were used to estimate the time to death from any cause after diagnosis of the invasive recurrence. The period of follow-up was cut off at December 31, 2020. A *p*-value < 0.05 was considered statistically significant. All statistical analyses were conducted using R software version 3.4.1. [5]

## Results

452 patients were identified with an invasive recurrence that occurred between 2000 and 2016, after initial treatment for DCIS between 1984 and 2014 with either BCS or mastectomy. Approximately half (54%, 244/452) were treated after the year 2000; 367 patients (81%) were initially treated with BCS and 85 patients (19%) were treated with mastectomy.

### DCIS characteristics at initial presentation

Clinicopathologic characteristics of patients with invasive recurrence after initial treatment with BCS vs. mastectomy are compared in Table 1. Patients who had undergone mastectomy were younger at initial diagnosis (median age 42 vs. 52 years, *p* < 0.001) and were more likely to have had high grade, multifocal or multicentric DCIS with necrosis (*p* < 0.001). Additionally, patients who had undergone mastectomy were more likely to have undergone axillary surgery than those in the BCS group (*p* < 0.001). Positive margins had been present in 11% of women who underwent BCS as compared with 5% of those treated with mastectomy (*p* = 0.07). In the BCS group, 20% had taken endocrine therapy vs. 13% after mastectomy (*p* = 0.33). 55% of patients who had undergone BCS had received radiation compared with 5% of those who had mastectomy. (*p* < 0.001). All patients with positive margins after mastectomy had received radiation.

**Table 1:** Clinicopathologic and treatment characteristics of initial DCIS in patients with invasive recurrence after BCS or mastectomy

	BCS (*n* = 367)	Mastectomy (*n* = 85)	*p*-value*
Age at initial surgery, median (range)	52 (22–88)	42 (21–70)	< 0.001
Era of initial treatment			
1984–2000	171 (47%)	37 (44%)	0.61
2001–2014	196 (53%)	48 (56%)	
Nuclear grade of DCIS			< 0.001
High	124 (34%)	49 (58%)	
Intermediate	126 (34%)	21 (24%)	
Low	45 (12%)	0 (0%)	
Unknown*	72 (20%)	15 (18%)	
Multifocal			< 0.001
Yes	53 (14%)	35 (41%)	
No	252 (69%)	36 (42%)	
Unknown*	62 (17%)	14 (17%)	
Multicentric			< 0.001
Yes	11 (3%)	32 (38%)	
No	291 (79%)	39 (46%)	
Unknown*	65 (18%)	14 (16%)	
Necrosis present			< 0.001
Yes	159 (43%)	49 (58%)	
No	152 (41%)	15 (18%)	
Unknown*	56 (16%)	21 (24%)	
Positive margins			0.07
Yes	41 (11%)	4 (5%)	
No	224 (61%)	60 (71%)	
Unknown*	102 (28%)	21 (24%)	
Axillary surgery			< 0.001
Yes	36 (10%)	61 (72%)	
No	331 (90%)	24 (28%)	
Endocrine therapy			0.33
Yes	74 (20%)	11 (13%)	
No	277 (76%)	61 (72%)	
Unknown*	16 (4%)	13 (15%)	
Radiation			< 0.001
Yes	201 (55%)	4 (5%)	
No	153 (42%)	68 (80%)	
Unknown*	13 (3%)	13 (15%)	

*Unknown categories are not considered in *p*-value calculation

Of our study population with invasive recurrence, all patients > 70 years (*n* = 28) had undergone BCS and only 29% of these (8/28) had received radiation (Table 2). Among the subset of women ≤ 70 years who had undergone BCS, 49% had received radiation (*p* = 0.05).

**Table 2:** Patients who had undergone initial BCS by age group and receipt of radiation

	Radiation	No radiation	*p*-value
Age group^a^			0.05
≤ 70 years	197 (49%)	201 (51%)	
> 70 years	8 (29%)	20 (71%)	

^a^Missing data in age ≤ 70 years (*n* = 24); age > 70 (*n* = 2)

### Invasive recurrence characteristics

Characteristics of invasive recurrences in patients who originally had BCS vs. mastectomy are compared in Table 3. Recurrence subtype was available for 92% (416/452) of patients, and histology was available for 87% (392/452). The majority of recurrences in all patients with known histology were infiltrating ductal carcinoma (86%; 337/392); the remaining 14% (55/392) were infiltrating lobular or other mixed mammary carcinomas (BCS: 13.5%; mastectomy: 18%), with a higher proportion of pure infiltrating lobular carcinomas in the BCS group (BCS: 8.4% vs. mastectomy: 0%; *p* < 0.001). Most recurrences were HR+/HER2− (71%, [296/416]); however, a higher proportion of HR-/HER2− recurrences were observed in the BCS group (11% vs 1.3%, *p* < 0.022). DCIS was documented in the biopsy or surgical specimen of the invasive recurrence in 67% (304/452) of cases. As expected, DCIS was more frequently noted in the recurrence specimen after initial BCS (285/314; 91%) than after initial mastectomy (19/30; 63%).

**Table 3:** Characteristics of first invasive recurrence after BCS or mastectomy for DCIS

	BCS (*n* = 367)	Mastectomy (*n* = 85)	*p*-value*
Recurrence subtype			0.022
HR+/HER2−	238 (70%)	58 (75%)	
HR+/HER2+	36 (11%)	11 (14%)	
HR−/HER2+	27 (8.0%)	7 (9.1%)	
HR−/HER2−	38 (11%)	1 (1.3%)	
Unknown*	28	8	
Histology			< 0.001
Ductal	290 (87%)	47 (82%)	
Lobular	28 (8.4%)	0 (0%)	
Other**	17 (5.1%)	10 (18%)	
Unknown*	32	28	
DCIS identified in recurrence			< 0.001
Yes	285 (91%)	19 (63%)	
No	29 (9.2%)	11 (37%)	
Unknown*	53	55	

*Unknown categories are not considered in *p*-value calculation

**Mixed ductal and lobular carcinoma

Of the 423 patients (94%) for whom information was available regarding receipt of endocrine therapy for DCIS, the majority of invasive recurrences were ER-negative (79%; 336/423). The proportion of ER-negative recurrences was not significantly different between patients who did and did not receive endocrine therapy (81% vs. 82%; *p* = 0.9) (Supplemental Table 1). Similarly, histology and nuclear grade of the recurrence was not significantly different between patients who did and did not take endocrine therapy.

**Fig. 1:**
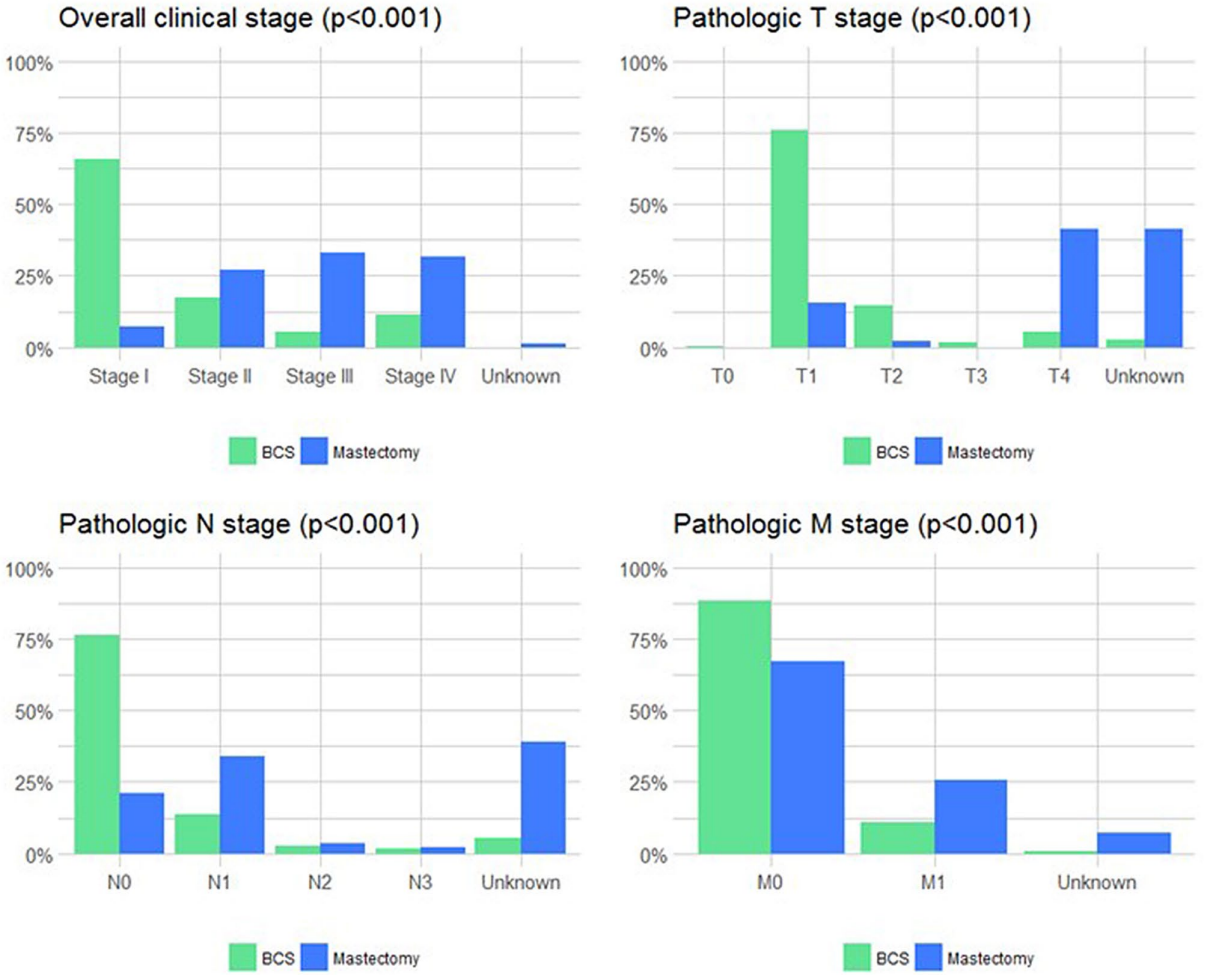
Clinical stage of recurrence among women presenting with invasive recurrence after BCS or mastectomy for DCIS

Of the 94% of patients treated with BCS who experienced an isolated local invasive recurrence, a significantly higher proportion were observed in the group that did not receive radiation compared to those who received radiation (98% [181/201] vs. 90% [162/166]; *p* = 0.005). Patients in the BCS group experienced a higher proportion of isolated local recurrences compared to the mastectomy group (94% vs. 12%; *p* < 0.001) (Table 3), which was also true of the subset of patients treated with BCS and radiation compared with mastectomy (90% vs. 12%; *p* < 0.001) (Supplemental Table 2). Regional or distant metastases were more frequent among those who had undergone initial mastectomy (88% mastectomy vs. 6.5% BCS; *p* < 0.001). Overall, recurrences after mastectomy presented more commonly as clinical stage II, III, or IV disease (*p* < 0.001) (Fig. 1). Among patients originally treated with mastectomy, pathologic T stage and nuclear grade of the recurrence was not significantly different between patients with DCIS noted in the recurrence specimen and those without DCIS noted (Supplemental Table 3).

### Time to invasive recurrence and overall survival

Overall there was no difference in time to recurrence between the mastectomy and BCS groups. However, median crude time to regional recurrence was longer in the BCS group compared with the mastectomy group (12.9 vs. 5.3 years, *p* = 0.03) (Table 4). After adjusting for initial DCIS characteristics and receipt of radiation in an accelerated failure time model, the time to regional recurrence was not statistically significantly different between surgical groups (*p* = 0.5). The presence of necrosis and multifocal disease were both significantly associated with regional recurrence in patients who received radiation (time ratio for necrosis: 0.7 [*p* = 0.04], time ratio for multifocality: 0.2 [*p* < 0.001]). We did not find any other significant associations between between type of surgery, or any other clinicopathologic factors, and time to local or distant recurrence.

**Table 4:** Patterns of invasive recurrence after treatment of DCIS by initial surgery type

	BCS* (*n* = 367)	Mastectomy (*n* = 85)	*p*-value
Presentation of invasive recurrence			< 0.001
Isolated local	343 (94%)	10 (12%)	
Local and regional	4 (1%)	5 (6%)	
Local and distant	8 (2%)	0 (0%)	
Local, regional and distant	2 (< 1%)	1 (1%)	
Isolated regional	1 (< 1%)	48 (56%)	
Regional and distant	5 (1%)	11 (13%)	
Isolated distant	4 (1%)	10 (12%)	
Median crude time to invasive recurrence, years			
All recurrences	6.4	5.5	0.12
Any local	6.3	7.4	0.62
Any regional	12.9	5.3	0.03
Any distant	7.0	5.9	0.78

*Includes patients who did and did not receive radiation

The overall survival (OS) of the entire study cohort from the time of diagnosis of the invasive recurrence was 55%, at a median follow-up of 6.7 years (range 0.1–21.4) (Supplemental Fig. 1) and was not statistically significantly different between surgical groups (*p* = 0.3).

## Discussion

In this cohort of patients who experienced a first invasive recurrence following treatment for DCIS, notable differences were identified between those who originally had undergone BCS vs. mastectomy. The majority of first recurrences in patients who underwent mastectomy were regional or distant, whereas isolated local recurrences were more common after BCS, particularly in those who did not receive radiation, as is expected. Thus, invasive recurrences among patients initially treated with BCS were more frequently clinical stage I compared to those originally treated with mastectomy.

Patients who developed an invasive recurrence after having undergone mastectomy had several characteristics associated with a higher risk profile compared with those who had undergone BCS, including younger age, higher grade DCIS with necrosis, and multifocal or multicentric disease. Young age is a known risk factor for locoregional recurrence (LRR) following both BCS and mastectomy for DCIS. In a contemporary cohort of patients treated with mastectomy for DCIS ± microinvasion, the cumulative 10-year incidence of LRR was 4.2% for patients age < 40 years, 2.0% for age 40–49 and 0.2% for age ≥ 50 years (*p* < 0.001) [6]. Similarly, in the setting of BCS, Cronin et al. reported that LRR risk decreased with age, in patients who did and did not receive radiation [7]. We found that young age contributed to the overall higher-risk profile of the mastectomy group relative to those who had BCS.

We cannot comment on the relationship between age, or any other prognostic factor, and risk of invasive recurrence in our cohort, which included only patients with invasive recurrences after treatment for DCIS and not all patients originally treated for DCIS. However, we did assess whether higher risk clinical features were associated with patterns of recurrence in patients treated with mastectomy vs. BCS. While initial surgical therapy was not associated with recurrence patterns, we found that the presence of necrosis and multifocal DCIS were significantly associated with shorter time to regional recurrence, after adjusting for initial surgery type and adjuvant treatment. A recent meta-analysis identified prognostic factors associated with ipsilateral invasive recurrence after DCIS, including age, high grade disease, and margin status. These were significant in spite of inclusion of two studies which included a substantial proportion of patients treated with mastectomy, who have lower risk of local recurrence compared with those treated with BCS [8–10]. Multiple clinical, pathologic, and treatment factors have been incorporated into a clinical risk estimation tool for use after BCS that has been externally validated in at least five independent populations [11–16], and can assist in decision-making regarding various treatment options for patients with DCIS. Unfortunately, the goal of more accurate risk assessment using multigene assays has not yet been achieved [17].

The predominance of isolated local recurrences of patients who had undergone BCS for DCIS is in part due to preservation of the breast, and additionally due to less aggressive original characteristics of their DCIS. Following BCS, there is a risk for persistence of unrecognized DCIS in the preserved ductal tissue, which in the absence of complete eradication by radiation, can progress to invasive disease. Theoretically, local invasive recurrences after mastectomy for DCIS result from incompletely resected ductal tissue containing DCIS or from unrecognized microscopic invasion present prior to mastectomy, however regional and distant recurrences are more likely related to more aggressive characteristics of the index disease or by the presence of microinvasive disease. High-grade DCIS has previously been associated with a histologically subtle loss of restricting basement membrane and increased risk of metastatic potential [18]. Furthermore, the presence of microinvasion in large mastectomy specimens may not have been consistently identified prior to standardization of pathologic protocols, given that a large proportion of patients in this series were initially treated two decades ago.

19 patients in our series initially treated with mastectomy experienced a locoregional invasive recurrence containing DCIS. 6 (32%) of these were classified in the medical record as regional recurrences; however, it is more likely that these represented local recurrences high in the axillary tail of the breast. This finding underscores the importance of completely excising all glandular tissue up to the superior and lateral anatomic borders of the breast to minimize retained breast tissue and LRR risk.

Differences in patterns of invasive recurrences between BCS and mastectomy patients may further be explained by differences in routine follow-up between surgical groups. Following BCS, patients are recommended to continue annual screening mammography, and therefore have the opportunity for prompt diagnosis of isolated local recurrence, including DCIS, prior to it becoming palpable. After mastectomy, although local recurrence is less common, the opportunity for early diagnosis through screening is not available and the recurrence is identified only after becoming palpable or involving the skin, thereby presenting as a higher T stage. In our study population, the majority of women who experienced a local recurrence after BCS had early T stage tumors, whereas a higher proportion of women with a local recurrence after mastectomy had T4 tumors and were more likely to present with synchronous regional or distant metastases. Following treatment with BCS for DCIS, National Comprehensive Cancer Network (NCCN) guidelines [18] recommend interval physical exam every 6–12 months for 5 years and annually thereafter; however, no guidelines exist regarding surveillance exam after mastectomy in this setting. In our series, the median crude time from initial treatment to invasive recurrence was approximately 6 years; therefore, clinical follow-up with annual physical examination is warranted, regardless of initial surgical therapy.

Proof of survival equivalency of BCS and mastectomy for invasive cancer led to adoption of BCS as an oncologically safe alternative to mastectomy for DCIS, where appropriate [19–23]. Overall survival is excellent with both treatment options; however, locoregional invasive recurrence rates vary by surgical treatment. In a meta-analysis of prospective and retrospective studies of patients with pure DCIS, the 10-year adjusted invasive LRR rate following mastectomy was 1.8% (95% CI 0.8–2.8%), 6.7% (95% CI 5.4–8.0%) in patients treated with BCS and radiation and 10.7% (95% CI 8.0–13.4%) in those treated with BCS alone (*p* < 0.001) [1]. No difference in overall or distant disease-free survival has been observed between different treatments [1, 24–26].

In our study population of women with a first invasive recurrence, the time to recurrence was similar between surgical groups; however, the predominant sites of recurrence were different, likely due to different risk profiles of the index disease between patients who had mastectomy vs. BCS. The overall survival (OS) of all patients who experienced an invasive recurrence was 55% and was not statistically significantly different between surgical groups, in spite of the greater proportion of regional and distant recurrences in patients initially treated with mastectomy. The low OS in our cohort is likely attributable to the long period of follow-up of our study, in which 47% (208/452) of patients were initially treated for their DCIS prior to the year 2000, as well as selection bias, as our cohort only included patients who went on to develop an invasive recurrence after their initial treatment for DCIS.

This study is the largest retrospective series, to our knowledge, of invasive recurrences following treatment for DCIS. Our study does have limitations, including its retrospective nature, and the fact that many patients were initially treated prior to routine incorporation of radiation in the DCIS treatment algorithm. As our cohort was comprised solely of patients with invasive recurrence after treatment for DCIS with BCS or mastectomy, we emphasize we cannot make inferences about the true incidence of invasive recurrence after treatment for DCIS, limiting the generalizability of our findings. Some patients were treated at other institutions for their initial DCIS, and a comprehensive record of disease and treatment characteristics was not available for review in some circumstances. Lastly, some patients with distant invasive recurrences did not undergo biopsy, and pathologic data on their recurrence was not available for review.

## Conclusions

We have demonstrated that the characteristics and patterns of invasive recurrence after DCIS vary significantly between patients initially treated with BCS vs. mastectomy. Patients who have undergone mastectomy are more likely to have high-risk features of their index disease and subsequently present with more advanced stage recurrences compared with patients treated with BCS. Clinical follow-up with annual physical examination is warranted after treatment for DCIS, regardless of initial surgical therapy.

## Supplementary Information

Below is the link to the electronic supplementary material.

Supplementary file1 (PDF 104 kb)
