# Supplementary material for: Patterns of invasive recurrence among patients originally treated for ductal carcinoma in situ by breast-conserving surgery versus mastectomy
Source: Breast Cancer Res Treat. 2021 Mar 6;186(3):617–24. doi: 10.1007/s10549-021-06129-3 (PMC8019411; doi:10.1007/s10549-021-06129-3)
Supplement: Supplementary file 1 — Supplementary file1 (PDF 104 kb) [file 10549_2021_6129_MOESM1_ESM.pdf]

Supplemental Figure 1. Overall survival of patients with an invasive recurrence after DCIS

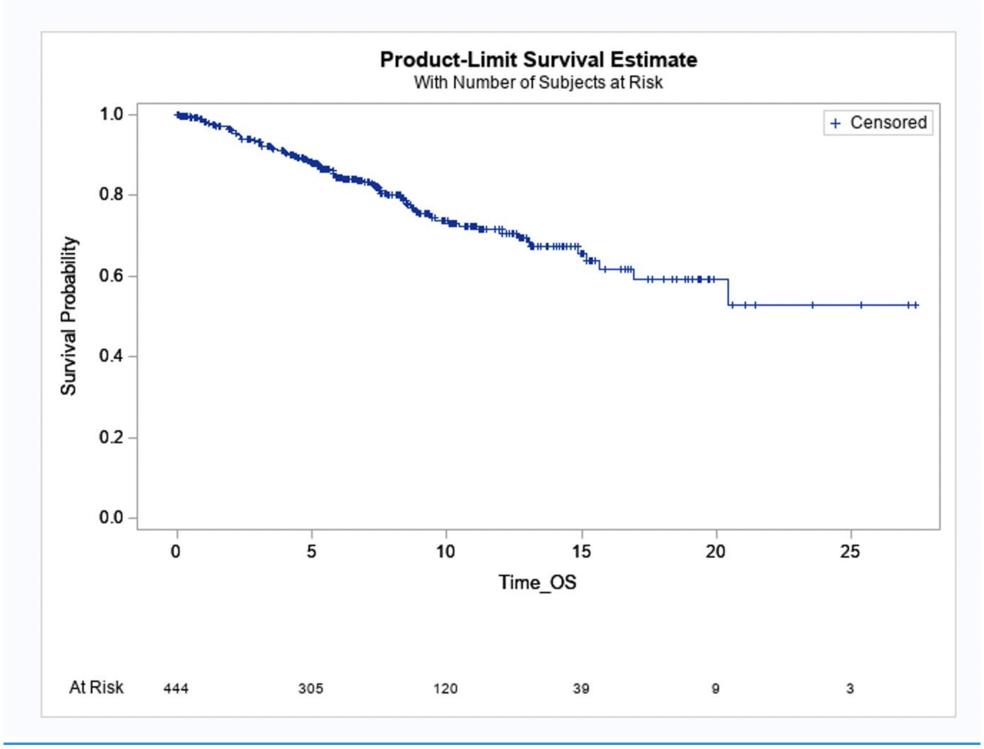

Supplemental Table 1. Comparison of invasive recurrence characteristics between patients who did and did not receive endocrine therapy

*Missing data on receipt of endocrine therapy for 29 (6%) of patients
**Unknown categories are not considered in p-value calculation

	**Endocrine* therapy (n = 85)**	**No endocrine therapy (n = 338)**	**p-value****
**ER status**			
Positive	15 (19%)	59 (18%)	0.9
Negative	65 (81%)	271 (82%)	
Unknown**	5	8	
**Histology**			
Ductal	67 (91%)	247 (84%)	0.5
Lobular	4 (5%)	24 (8%)	
Other	3 (4%)	22 (8%)	
Unknown**	11	45	
**Nuclear grade**			
High	34 (46%)	121 (46%)	0.5
Intermediate	38 (51%)	127 (48%)	
Low	2 (3%)	17 (6%)	
Unknown**	11	73	

Supplemental Table 2. Patterns of invasive recurrence after treatment of DCIS among patients treated with BCS and radiation vs. mastectomy

*Missing data for date of diagnosis of distant recurrence in BCS + radiation group

	**BCS + radiation (n = 201)**	**Mastectomy (n = 85)**	**p-value**
**Presentation of invasive recurrence**			<0.001
Isolated local	181 (90%)	10 (12%)	
Local and regional	1 (<1%)	5 (6%)	
Local and distant	8 (4%)	0 (0%)	
Local, regional and distant	2 (1%)	1 (1%)	
Isolated regional	1 (<1%)	48 (56%)	
Regional and distant	4 (2%)	11 (13%)	
Isolated distant	4 (2%)	10 (12%)	
**Median crude time to invasive recurrence, years**			
All recurrences	6.3	5.5	0.24
Any local	7.0	7.4	0.63
Any regional	5.9	5.3	0.05
Any distant*	-	5.9	n/a

Supplemental Table 3. Comparison of pathologic T stage and nuclear grade of the invasive recurrence between patients originally treated with mastectomy with and without DCIS noted in the recurrence specimen

*Missing data on DCIS in recurrence for 108 (24%) of patients
**Unknown categories are not considered in p-value calculation

	**DCIS in recurrence specimen* (n = 19)**	**No DCIS in recurrence specimen (n = 66)**	**p-value****
**Pathologic T stage**			0.3
T1	6 (35%)	7 (16%)	
T2	0 (0)	2 (4.7%)	
T3	0 (0)	0 (0)	
T4	10 (59%)	25 (58%)	
Tx	1 (5.9%)	9 (21%)	
Unknown**	2	23	
**Nuclear grade**			
High	11 (69%)	18 (72%)	0.8
Intermediate	5 (31%)	6 (24%)	
Low	0 (0)	1 (4%)	
Unknown**	3	41	
